# Supplementary material for: Resolving the full spectrum of human genome variation using Linked-Reads
Source: Genome Res. 2019 Apr;29(4):635–45. doi: 10.1101/gr.234443.118 (PMC6442396; doi:10.1101/gr.234443.118)
Supplement: Supplemental Material [file supp_gr.234443.118_Supplemental_longranger-2.2.zip › longranger-2.2.tar.bz2!/lr2.2-public/lib/stan/cmdstan/stan/doc/api/html/math_2matrix_2qr___q_8hpp_source.html]

Stan: src/stan/math/matrix/qr\_Q.hpp Source File


|  |  |
| --- | --- |
|  | Stan  2.5.0  probability, sampling & optimization |


- Main Page
- Related Pages
- Namespaces
- Classes
- Files

- File List
- File Members

All Classes Namespaces Files Functions Variables Typedefs Enumerations Enumerator Friends Macros Pages

- src
- stan
- math
- matrix

qr\_Q.hpp

Go to the documentation of this file.

1 #ifndef STAN\_\_MATH\_\_MATRIX\_\_QR\_Q\_HPP

2 #define STAN\_\_MATH\_\_MATRIX\_\_QR\_Q\_HPP

3

4 #include <stan/math/error\_handling/check\_greater\_or\_equal.hpp>

5 #include <stan/math/error\_handling/matrix/check\_nonzero\_size.hpp>

6 #include <stan/math/matrix/Eigen.hpp>

7 #include <Eigen/QR>

8

9 namespace stan {

10  namespace math {

11

12  template <typename T>

13  Eigen::Matrix<T,Eigen::Dynamic,Eigen::Dynamic>

14  qr\_Q(const Eigen::Matrix<T,Eigen::Dynamic,Eigen::Dynamic>& m) {

15  typedef Eigen::Matrix<T,Eigen::Dynamic,Eigen::Dynamic> matrix\_t;

16  stan::math::check\_nonzero\_size("qr\_Q(%1%)",m,"m",(double\*)0);

17  stan::math::check\_greater\_or\_equal("qr\_Q(%1%)",static\_cast<size\_t>(m.rows()),

18  static\_cast<size\_t>(m.cols()),"m.rows()",

19  (double\*)0);

20

21  Eigen::HouseholderQR<matrix\_t> qr(m.rows(), m.cols());

22  qr.compute(m);

23  matrix\_t Q = qr.householderQ();

24  for (int i = 0; i < m.cols(); i++)

25  if (qr.matrixQR()(i,i) < 0)

26  Q.col(i) \*= -1.0;

27  return Q;

28  }

29

30  }

31 }

32 #endif

stan::math::qr\_Q

Eigen::Matrix< T, Eigen::Dynamic, Eigen::Dynamic > qr\_Q(const Eigen::Matrix< T, Eigen::Dynamic, Eigen::Dynamic > &m)

**Definition:** qr\_Q.hpp:14

Eigen.hpp

check\_greater\_or\_equal.hpp

check\_nonzero\_size.hpp

stan::math::check\_greater\_or\_equal

bool check\_greater\_or\_equal(const char \*function, const T\_y &y, const T\_low &low, const char \*name, T\_result \*result)

**Definition:** check\_greater\_or\_equal.hpp:58

stan::math::check\_nonzero\_size

bool check\_nonzero\_size(const char \*function, const T\_y &y, const char \*name, T\_result \*result)

Return true if the specified matrix/vector is of non-zero size.

**Definition:** check\_nonzero\_size.hpp:33


---

[ Stan Home Page ]

*© 2011–2014,
Stan Development Team.*
